# A convolutional neural network for fully automated total metabolic tumor volume delineation in patients with aggressive Non-Hodgkin lymphoma

**DOI:** 10.1007/s00259-026-07810-9

**Published:** 2026-03-24

**Authors:** Pavel Nikulin, Sebastian Hoberück, Ivayla Apostolova, Jens Maus, Andreas Hüttmann, Ulrich Dührsen, Frank Kroschinsky, Jörg Kotzerke, Malte von Bonin, Ralph A. Bundschuh, Anja Braune, Frank Hofheinz

**Affiliations:** 1 Department of Positron Emission Tomography, Helmholtz-Zentrum Dresden-Rossendorf, Institute of Radiopharmaceutical Cancer Research, Dresden, Germany; 2 Department of Nuclear Medicine, University Hospital Carl Gustav Carus, Technische Universität Dresden, Dresden, Germany; 3 Department of Diagnostic and Interventional Radiology and Nuclear Medicine, University Hospital Hamburg-Eppendorf, Hamburg, Germany; 4 Department of Hematology, West German Cancer Center, University Hospital Essen, University of Duisburg-Essen, Essen, Germany; 5 Medical Department I, Dresden University Hospital, Dresden, Germany; 6 Helmholtz-Zentrum Dresden-Rossendorf, Institute of Radiopharmaceutical Cancer Research, Dresden, Germany; 7 German Cancer Consortium (DKTK), Partner Site Dresden, Dresden, Germany; 8 National Center for Tumor Diseases (NCT), NCT/UCC Dresden, a partnership between DKFZ, Faculty of Medicine and University Hospital Carl Gustav Carus, TUD Dresden University of Technology, and Helmholtz-Zentrum Dresden-Rossendorf, Dresden, Germany; 9 Carl Gustav Carus Faculty of Medicine, Technische Universität Dresden, Dresden, Germany

**Keywords:** FDG PET, Total metabolic tumor volume, TMTV, Non-Hodgkin lymphoma, Convolutional neural network, Delineation

## Abstract

**Purpose:**

The [^18^F]FDG-PET-derived total metabolic tumor volume (TMTV) has a high prognostic value in patients with Hodgkin and Non-Hodgkin lymphoma. However, in order to enable TMTV as a biomarker for clinical use, an accurate and fast method of tumor delineation in lymphoma patients is needed. Deep-learning-based methods have shown promising results in this field and offer distinct advantages over classical approaches. Therefore, the goal of this work was to train a convolutional neural network (CNN) for delineation of all lymphoma lesions regardless of their size and uptake characteristics while performing the optimal contouring of each individual lesion.

**Methods:**

A neural network was trained with the nnU-Net software package. A total of 1192 [^18^F]FDG-PET/CT scans from 716 patients with Non-Hodgkin lymphoma participating in the PETAL trial comprised the main dataset which was used for training. The ground truth delineation included all lesions that were clinically considered as lymphoma manifestations by an experienced observer and was developed iteratively with the assistance of intermediate CNN models. Performance of the trained network was assessed in the main dataset via 5-fold cross-validation as well as in external benchmark dataset (*N* = 60 scans).

**Results:**

Comparing the manual and automated delineations in the main (external) dataset, the aggregated Dice coefficient reached 0.895 (0.715) and the corresponding TMTVs were highly correlated with *R*^2^ = 0.974 (0.767). The main (external) dataset contained a total of 8971 (713) manually delineated lesions, the detection sensitivity of which was 71.2% (77.6%) with the positive predictive value of 84.1% (63.1%). Univariate Cox regression analysis in the main dataset revealed both manually and automatically derived TMTVs as highly prognostic factors for progression-free survival with very similar hazard ratios (HR = 3.5; p < 0.001 and HR = 3.7; *p* < 0.001, respectively).

**Conclusion:**

In this study we presented a CNN model capable of accurate TMTV delineation in [^18^F]FDG-PET/CT images of lymphoma patients. It is trained to delineate all tumor lesions while accounting for typical caveats inherent in this task. The developed neural network allows for substantial acceleration of quantitative analysis of lymphoma imaging data and has the potential for supervised clinical use.

**Supplementary Information:**

The online version contains supplementary material available at 10.1007/s00259-026-07810-9.

## Introduction

Despite modern immunochemotherapy tremendously improving the prognosis of aggressive B-cell Non-Hodgkin lymphoma, around one third of the patients experience a refractory or relapsing disease [1]. Therefore, a biomarker for identifying these patients is urgently needed. Such a biomarker could be derived from [^18^F]FDG-PET imaging (hybrid with CT or MRI), which is nowadays considered the state of the art in primary staging and response assessment of aggressive lymphoma [2–5]. For example, in the PETAL trial [6], early therapy response was evaluated by [^18^F]FDG-PET/CT using a 66% decline of the SUV_max_ between the initial and the interim scans. An alternative biomarker could be the [^18^F]FDG-PET-derived total tumor burden or total metabolic tumor volume (TMTV), which has a high prognostic value in patients with Non-Hodgkin [7–14] and Hodgkin lymphoma [15]. However, in these studies, TMTV was determined from threshold-based tumor delineations with either absolute (e.g., SUV = 4.0) or relative (e.g., 41% of SUV_max_) thresholds. It is well known that the threshold-based delineation methods are susceptible to variations in image and tumor characteristics, leading to under- or over-estimation of TMTV [16, 17] either sporadically (patient-dependent) or systematically (scanner- and reconstruction-dependent). These inconsistencies may lead to a reduction in the prognostic value of TMTV and hamper the reproducibility of the established TMTV thresholds for risk-stratification [18]. Moreover, these methods require manual classification of the delineated uptake into pathological and physiological, which can be very time consuming. Therefore, in order to enable TMTV as a biomarker for routine clinical use, a robust and fast method of tumor delineation in lymphoma patients is needed.

A potential candidate for such a method is an AI-assisted lesion delineation. Based on the degree of such assistance, one can differentiate between hybrid and fully Deep Learning (DL) approaches. Hybrid approaches rely on classical algorithms for the detection and delineation of [^18^F]FDG-avid uptake regions and employ a Convolutional Neural Network (CNN) for their classification into suspicious or non-suspicious for cancer. The most prominent solution of this kind is the PET-Assisted Reporting System (PARS, Siemens Medical Solutions USA, Inc., Malvern, PA, USA), which combines the CNN proposed in [19] and the Multi-Foci Segmentation (MFS) algorithm aligned with PERCIST recommendations [20]. PARS was independently evaluated in multiple cohorts of Diffuse Large B-Cell Lymphoma (DLBCL) patients, showing only moderate agreement with the manual delineation in terms of spatial overlap and TMTV correlation, leading to an actual reduction in TMTV’s prognostic value [21–23]. Purely DL methods, on the other hand, have the potential to overcome the limitations of classical contouring algorithms by incorporating the knowledge that an experienced observer put into producing the ground truth delineations. These methods typically rely on large, sometimes encompassing multiple cancer types [24], datasets for the network training (up to 2270 scans in [25]), which are necessitated by the high variability of uptake patterns in the different lymphoma subtypes. State-of-the-art methods normally feature CNNs with U-Net-like architectures, either alone [26–28] or in cascade [24, 25]. Additions, like anomaly detection via generative adversarial networks [29] or evidential segmentation layers [30], show promising results, potentially boosting the performance of such networks even further. Overall, pure DL-based solutions demonstrate high concordance with the manual delineations in terms of spatial overlap and TMTV correlation [25, 27]. High concordance between delineations should also lead to similar results in survival analysis based on these delineations, but — to the best of our knowledge — it has not been demonstrated yet. For further details on the use of AI in lymphoma imaging, we refer to [31, 32].

Despite the potential of DL approaches to overcome the inherent flaws of classical delineation methods, the resulting network’s performance depends crucially on the ground truth data used for the training. So far, however, the ground truth delineations were mainly performed using absolute or relative threshold methods with the same threshold applied to all lesions and manual corrections being limited to inclusion or exclusion of PET-positive foci [25–28]. Therefore, the full potential of neural networks to adapt to different imaging conditions and lesion properties has not been realized yet. A step in this direction has been taken with the introduction of the AutoPET challenge [24] and the corresponding PET/CT dataset with free-hand lesion delineations [33]. However, the number of lymphoma patients included in this dataset is quite limited (*N* = 145 scans). Moreover, fully manual delineation might have higher interobserver variability than semi-automatic methods [34], the consequences of which for the neural network training are not well understood yet. Additionally, small lesions (< 1 ml) are often excluded from the ground truth delineations [35, 36], while their actual influence on the prognostic value of TMTV, as well as other potential biomarkers, is so far unclear [17, 18]. Therefore, the goal of this work was to train a CNN for delineation of all lymphoma lesions regardless of their size and uptake characteristics while performing the optimal contouring of each individual lesion. Additionally, we estimated the potential impact of including small lesions on extracted TMTV values.

## Methods

### Patients and data

The data used for this study comprised two datasets: the main dataset for the network development and internal evaluation, and the external dataset for independent evaluation of the network’s performance. The main dataset consisted of patient scans acquired as part of the PETAL trial [6]. Within the PETAL trial, *N *= 1073 patients were screened, of whom *N *= 862 were actually included. The included patients underwent a baseline [^18^F]FDG-PET scan prior to therapy and an interim PET scan after 2 cycles of chemotherapy. In the current study, the main dataset included PET/CT scans of the patients who actually entered the PETAL trial (baseline and interim scans) and for whom the CT used for attenuation correction was available. Out of these, *N *= 42 scans had to be excluded due to the low image quality (excessively high image noise or severe reconstruction artifacts resulting in loss of the signal from parts of the image), resulting in *N* = 1030 scans from *N* = 592 patients being included. To improve the performance of the network, we additionally included data with rather complex delineation tasks (many small lesions, bulky lesions, large lesions with diffuse tracer accumulation, etc.) from patients who were not eligible for inclusion in the PETAL trial (*N* = 162 scans). Altogether, *N *= 1192 PET/CT datasets from *N* = 716 patients were included in the current study (414 men, 302 women, age 61.2 ± 21.9 years). The data were acquired at *N* = 47 participating centers. Details on data acquisition and image reconstruction can be found in [6] and citations therein. Details on histology, staging and therapy are listed in supplementary Table S1.

As an external dataset we used the recently proposed benchmark for TMTV delineation in lymphoma patients [18]. The benchmark consists of *N* = 60 [^18^F]FDG-PET/CT scans of patients with different lymphoma subtypes (DLBCL, Hodgkin, and follicular lymphoma, *N* = 20 scans each), accompanied by expert annotations. The scans were chosen to challenge the delineation algorithms and are not representative of the general population. More details are provided in the respective publication.

### Ground truth definition

In the main dataset, all lesions which were clinically considered as lymphoma by an experienced observer, regardless of their volume or maximum uptake, were included in the ground truth delineations. The spleen was considered PET-positive whenever uni- or multi-focal uptake or inhomogeneous uptake with components above hepatic background was present. Homogeneous uptake, even in the context of splenomegaly and an uptake level above hepatic background, was considered benign.

Delineation of all lesions with classical methods is an extremely time-consuming task, especially in patients with many lesions. To accelerate the ground truth generation, we therefore employed an iterative network training approach, where an initial training was done with only a small subset of the available data and the developed models assisted in labeling further portions of training data. Semi-automated delineations of initial data, as well as corrections of CNN-generated labels, were performed using the software ROVER (version 3.0.78, ABX GmbH, Radeberg, Germany) by a tandem of a nuclear medicine physician (SH) with over 10 years of experience in oncological PET and a physicist (FH) with over 20 years of experience in the development of delineation algorithms in PET. A detailed description of the iterative procedure, choice of the semi-automatic delineation algorithms, and the illustration of the development of the network’s performance through iterations is presented in the supplementary material.

In the external dataset, the included delineations were used as the ground truth. These delineations were generated with the semi-automated threshold-based (SUV = 4) method, considering a minimum lesion volume of 3 ml, and were corrected manually for false-negative and false-positive findings. For more details, see [18].

We will refer to the ground truth delineations as "manual" further in the manuscript.

### Network training and inference

The network design and training were performed with the nnU-Net (version 2.5) [37] software framework for Python (version 3.10) based on PyTorch (version 2.3.1) DL library. The details on the training procedure as well as the utilized options, configurations, and the rationale behind their choice are given in the supplementary material. Shortly, the training was performed with three network configurations in each iteration (except for the first one). During the iterative training procedure, all processed data were used for training in each step. In the final iteration, the data was split into training and testing subsets in a proportion of 80/20% for the 5-fold cross-validation procedure (see below). CT and PET images served as the networks’ inputs, and the manual delineations served as the ground truth for the training.

For the inference (prediction) procedure, the outputs of all three (two in the first iteration) developed network models corresponding to the aforementioned nnU-Net configurations were combined. Inference followed the default nnU-Net procedure without further modifications. The outputs of the individual networks were combined after their binarization via the union operation. Union operation, compared to e.g. majority voting or output probability averaging, allows an increase in sensitivity at the expense of positive predictive value, bringing these two metrics into a better balance. Further in the manuscript, the network output will always refer to the combined output of all available network models.

### Network evaluation

For the final evaluation of the developed networks in the main dataset, the 5-fold cross-validation scheme was employed. For this, the main dataset (*N* = 1192 scans) was quasi-randomly split into 5 subsets (folds). During the split, it was ensured that all the scans of a single patient were set to the same fold to avoid cross-contamination. Additionally, the folds were roughly balanced in terms of patient-wise TMTV distributions. The network training was repeated 5 times (for each configuration), each time using a different fold as a testing set and the union of the rest of the folds as a training set. The developed models were used to produce delineations in the respective training sets according to the inference procedure described in the supplementary material. Finally, predictions for all 5 folds were pulled together for the final evaluation.

In the external dataset, the inference was performed with all 15 models developed for each respective fold and each network configuration. The ensembling was done first across the folds with the default nnU-Net procedure (averaging predicted voxel class probabilities followed by binarization) and then across configurations as described above (union operation).

The postprocessing consisted of cropping the image volumes by 5 mm on either side in the axial direction (to remove often-present artifacts) and excluding all lesions with volumes < 0.1 ml in both manual and automated delineations.

The automated delineations were assessed in terms of spatial concordance, lesion detection capabilities, and survival analysis utility using manual delineations as the reference. Results for both the cross-validation study and the external validation are reported. Non-pathological scans were excluded from spatial concordance and lesion detection analysis to put the focus on relevant cases. False-positive findings in these patients were reported separately. The analysis was conducted in the R language and environment for statistical computing (version 4.5.1) [38].

### Spatial concordance

The spatial concordance between automated and manual delineation was assessed using the Dice Similarity Coefficient (DSC). Both, image-wise and aggregated (considering all image volumes at once [39, 40]) DSCs were calculated. Mean, standard deviation (SD), median, and interquartile range (IQR), as well as the histogram for the image-wise DSC distribution, were reported. The volumetric concordance was evaluated in terms of TMTV. Correlation between manual and automated TMTVs (TMTV_man_ and TMTV_cnn_, respectively) was presented as a scatter plot and evaluated numerically with the square of the Pearson correlation coefficient *R*^2^. The top 1% outliers in terms of absolute TMTV deviation were excluded from correlation analysis for stability. More details on justification of the selected cutoff value are provided in the supplementary material. We also report on the robust to outliers Spearman correlation coefficient *ρ* without exclusion of any data. Additionally, the distribution statistics for (absolute) TMTV difference between the two delineations (ΔTMTV = TMTV_cnn_ − TMTV_man_) were presented, and a comparison between TMTV_man_ and TMTV_cnn_ was visualized in the Bland-Altman plot.

We have also evaluated the potential impact of exclusion of small lesions on the TMTV determination. For all images in the main dataset, the lesions below the specified variable volume threshold were excluded and the resulting TMTV difference ΔTMTV_excl _was calculated. We report on maximal recorded ΔTMTV_excl_ for each threshold value between 0.1 ml and 3 ml, as well as on the average within the top 10% quantile of ΔTMTV_excl_ distribution.

### Lesion detection

The methodology for lesion detection analysis follows [41] and was employed by us before in [40]. Briefly, for each lesion in manual and automated delineations, the coverage fraction by the complimentary delineation is computed. The lesions, for which the coverage fractions exceeded the 10% threshold, were considered “detected”. The detected and undetected lesions in manual delineation were denoted true positive with respect to the manual delineation (TP_man_) and false negative (FN), respectively. Analogously, the detected and undetected lesions in automated delineation were denoted true positive with respect to the automated delineation (TP_cnn_) and false positive (FP), respectively. Then, the true positive rate of lesion detection, also known as sensitivity, can be defined as TPR = TP_man_/(TP_man_+ FN) and the positive predictive value as PPV = TP_cnn_/(TP_cnn _+ FP). F_1_-score was used as a balanced metric of detection performance, defined as the harmonic mean of TPR and PPV or F_1_ = 2 × TPR × PPV/(TPR + PPV).

For better insight into the lesion detection performance of the CNN, the summary statistics for all lesions were complemented by the lesion-volume-stratified statistics. For this, lesions were binned into four categories according to their volumes: [0.1 ml, 1 ml), [1 ml, 10 ml), [10 ml, 100 ml), and [100 ml, ∞ ml). Note that manually and automatically defined lesions were stratified independently and were used to calculate the respective TPR and PPV. The coverage fractions were not recalculated after the stratification.

### Survival analysis

Finally, we investigated the impact of the differences between manual and automated delineation on the survival analysis. The full survival analysis is beyond the scope of this publication, therefore, we have only exemplarily considered the prognostic value of TMTV for progression-free survival (PFS). All patients from the main dataset with available baseline PET/CT and clinical follow-up information were considered eligible. A total of *N *= 534 patients were included in the analysis. The cutoff for the definition of high/low-risk groups was determined by minimizing the *p*-value in univariate Cox regression. The optimal cutoff was determined for TMTV_man_ and then applied to both TMTV_man_ and TMTV_cnn_. Prognostic value was assessed via univariate Cox regression and Kaplan-Meier analysis. Results with *p* < 0.05 were considered significant.

## Results

485 images did not show any pathological uptake and were excluded from the analysis of delineation and lesion detection performance. Out of these, 178 scans were falsely identified as pathological by the CNN. In total, 463 false positive lesions were identified in these scans, with a mean (median) volume of 7.59 ml (0.59 ml). The most frequent occurrences of the false positive uptake identification were associated with the elevated tracer concentration at injection site, in ureter, and in the intestine. The remaining 707 images were analyzed in more detail.

**Fig. 1:**
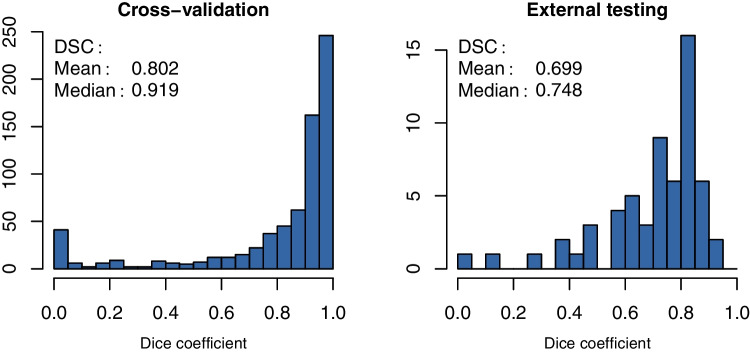
Frequency distribution of the observed Dice coefficients (CNN vs. manual delineation/labeling) in the cross-validation (left, *N* = 707 images) and external testing (right, *N* = 60 images)

**Fig. 2:**
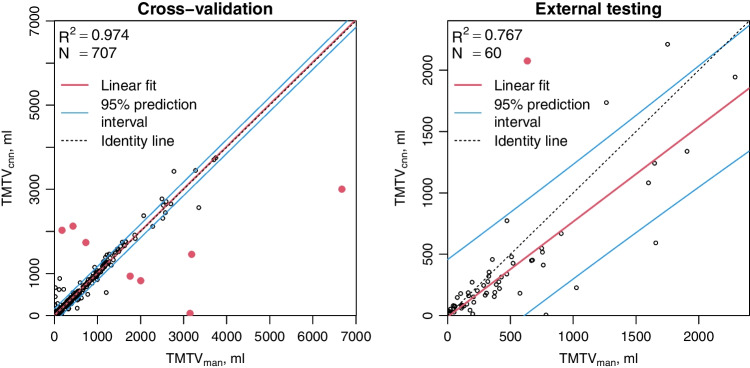
Correlation between manually and automatically derived TMTV in the cross-validation (left, *N* = 707 images) and external testing (right, *N *= 60 images) data. Solid red points indicate outliers, defined as data points where the deviation of CNN from manual delineation exceeds the 99% percentile (i.e. the top 1%). These outliers were excluded from the regression analysis. The red line represents the least squares fit of a straight line to the remaining data. The blue lines delineate the corresponding 95% prediction (tolerance) interval of the expected scatter of individual data points around the regression line

Exclusion of PET-negative scans has the aim of focusing the evaluation on the relevant cases, i.e. PET-positive lymphoma patients which actually require assistance in delineation. Importantly, our CNN was not optimized for screening purposes and is expected to perform poorly in this task. For completeness, we provide versions of Tables 1 and 2 as well as Figs. 1 and 2 for the whole main dataset without exclusions in the supplementary material.

### Spatial concordance

**Table 1:** Delineation performance in cross-validation and external testing data measured by different metrics. *N* indicates the number of images included in the analysis

Metric	Aggregated	Mean ± SD	Median	IQR
Cross-validation (*N* = 707)				
DSC	0.895	0.802 ± 0.269	0.919	[0.787, 0.961]
TPR	0.896	0.821 ± 0.267	0.941	[0.806, 0.980]
PPV	0.895	0.838 ± 0.269	0.947	[0.857, 0.980]
External testing (*N *= 60)				
DSC	0.715	0.699 ± 0.188	0.748	[0.635, 0.824]
TPR	0.633	0.693 ± 0.243	0.714	[0.578, 0.875]
PPV	0.821	0.829 ± 0.201	0.939	[0.681, 0.990]

**Table 2:** TMTV determination performance in cross-validation and external testing data measured by different metrics. *N* indicates the number of images included in the analysis

Metric	Cross-validation (*N* = 707)			External testing (*N* = 60)		
	Mean ± SD	Median	IQR	Mean ± SD	Median	IQR
TMTV_man_ (ml)	302.6 ± 575.8	79.0	[10.3, 339.6]	483.8 ± 527.7	323.4	[117.2, 643.1]
TMTV_cnn_ (ml)	298.2 ± 525.8	78.9	[10.9, 338.0]	392.0 ± 516.7	196.1	[80.8, 449.6]
$\Delta$TMTV (ml)	−4.4 ± 238.0	-0.3	[-6.1, 4.4]	−91.8 ± 324.7	-50.4	[-194.3, 12.5]
$$\big {|}$$$$\Delta$$TMTV$$\big {|}$$(ml)	47.8 ± 233.2	5.3	[1.2, 19.3]	198.7 ± 271.8	100.6	[32.3, 226.6]

**Fig. 3:**
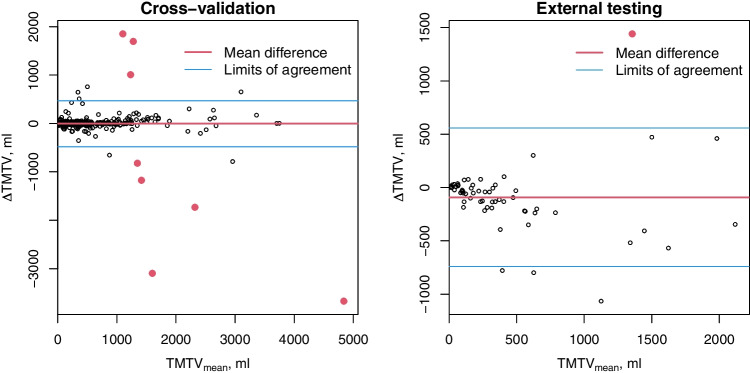
Bland-Altman plots for manually and automatically derived TMTV in the cross-validation (left, *N* = 707 images) and external testing (right, *N *= 60 images) data. Solid red points indicate the same outliers as in Fig. 2, however, they were not excluded from this analysis. The blue lines delineate the limits of agreement defined as mean ± 1.96 SD

**Fig. 4:**
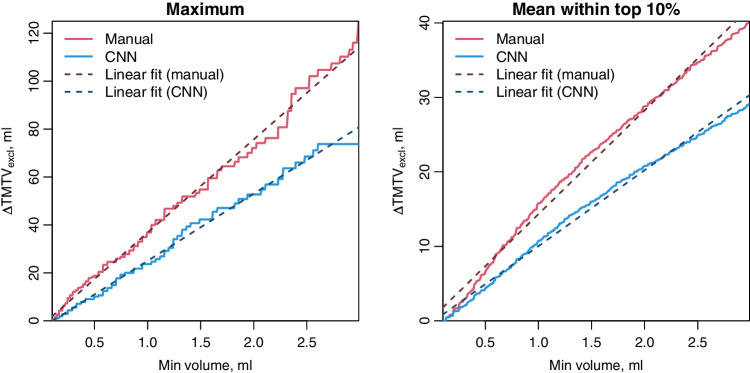
The maximum (left) and the average within the top 10% percentile (right) of observed TMTV differences between the ground truth delineations and the same delineations with small lesions (below the specified volume threshold) excluded

Summary statistics for delineation performance in the main and external datasets, respectively, are shown in Table 1. In the cross-validation, the aggregated DSC has reached 0.895 with a very good balance between the TPR and the PPV on the voxel level (0.896 vs 0.895 for respective aggregated metrics). The values were lower in the external dataset with 0.715, 0.633, and 0.821 for DSC, TPR, and PPV, respectively.

The distribution of Dice coefficients per image had a median of 0.919 and an IQR of [0.787, 0.961] in the main dataset, and a median of 0.748 and an IQR of [0.635, 0.824] in the external dataset. The corresponding histograms of the observed Dice coefficients are displayed in Fig. 1. Note the non-Gaussian shape of the distributions.

The correlation between manually and automatically derived TMTVs is shown in Fig. 2. After exclusion of the outliers, the volumes were highly correlated in the main dataset (*R*^2^ = 0.974, *ρ* = 0.965) and reasonably correlated in the external dataset (*R*^2 ^= 0.767, *ρ*^2 ^= 0.837). The summary statistics on the TMTV determination performance are given in Table 2. The mean (median) TMTV_man_ and TMTV_cnn_ were, respectively, 302.6 ml (79.0 ml) and 298.2 ml (78.9 ml) in the main dataset and 483.8 ml (323.4 ml) and 392.0 ml (196.1 ml) in the external dataset. The TMTV determination bias was −4.4 ml and −91.8 ml in the main and external datasets, respectively, while the mean absolute error of the TMTV determination was 47.8 ml and 198.7 ml. The relation between the TMTV difference and the average TMTV between the two delineations is shown in the Bland-Altman plot in Fig. 3.

Figure 4 demonstrates the extent of potential errors in TMTV determination caused by excluding small lesions from the analysis. Both, the maximal error and average top 10% error are growing nearly linearly with the increase of the lesion exclusion threshold in the chosen volume range. The maximal error in TMTV determination reached over 120 ml for lesions with volumes below 3 ml being excluded from manual delineation. Note, that exclusion of small lesions shows a bigger impact in manual delineations compared to automated ones, indicating potential deficiency in small lesion detection by CNN, which is confirmed in the lesion detection analysis.

### Lesion detection

**Table 3:** Detection performance in cross-validation and external testing data stratified by lesion volume. N_man_ and N_cnn_ denote the number of individual lesions in manual and automated delineations, respectively, and *N* indicates the number of images included in the analysis

Volume range	N_man_	N_cnn_	TPR	PPV	F_1_
Cross-validation (*N* = 707)					
[0.1 ml, 1 ml)	4491	3823	56.0%	76.6%	0.647
[1 ml, 10 ml)	3103	2559	82.9%	90.3%	0.865
[10 ml, 100 ml)	1020	963	92.8%	92.9%	0.929
[100 ml,∞)	357	355	97.8%	96.6%	0.972
Total	8971	7700	71.2%	84.1%	0.771
External testing (*N* = 60)					
[0.1 ml, 1 ml)	169	490	63.9%	44.1%	0.522
[1 ml, 10 ml)	352	326	76.4%	74.2%	0.753
[10 ml, 100 ml)	142	133	91.5%	94.7%	0.931
[100 ml,∞)	50	42	92.0%	97.6%	0.947
Total	713	991	77.6%	63.1%	0.696

The mean (median) lesion volume in manual and automated delineations was, respectively, 23.85 ml (0.99 ml) and 27.38 ml (1.02 ml) in the main dataset and 40.71 ml (3.91 ml) and 23.74 ml (1.02 ml) in the external dataset. The overall TPR and the PPV of lesion detection were, respectively, 71.2% and 84.1% in the main dataset and 77.6% and 63.1% in the external dataset. The detailed breakdown of lesion detection performance by the individual lesion volume is given in Table 3. In both datasets, the lesion detection performance increased with the lesion size, and for lesions over 10 ml in volume, both TPR and the PPV exceeded 90%. The relatively low values of the overall lesion detection metrics are explained by the prevalence of the small lesions (< 1 ml) for which the detection remains challenging (TPR of 56.0% and 63.9% in the main and external datasets, respectively). In general, human readers identified more lesions than the CNN in all cases except for the lesions < 1 ml in size in the external dataset, for which the manual delineation contained 169 lesions vs. 490 lesions in the automated delineation.

### Examples

**Fig. 5:**
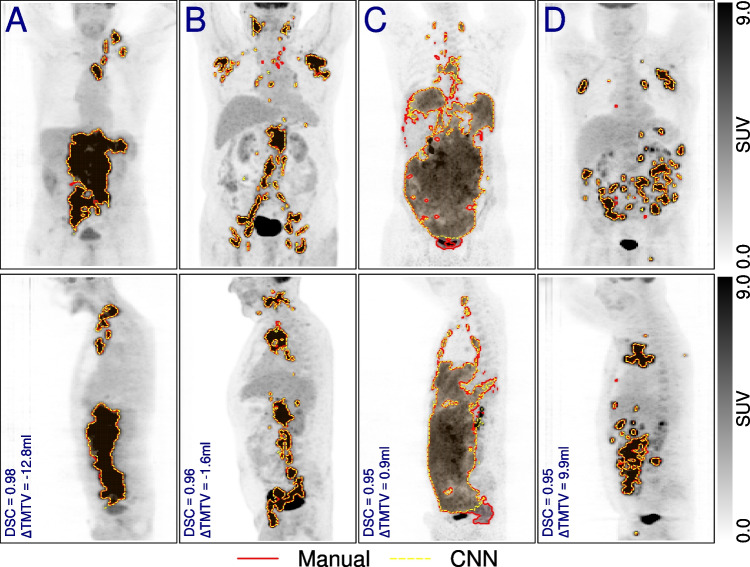
Visualization of successful automated delineations in four exemplary studies (**A**-**D**) from the test subsets of the cross-validation experiment. Shown are coronal from anterior (top) and sagittal (bottom) maximum intensity projections of two Stage III (**A** & **B**) and two Stage IV (**C** & **D**) lymphoma patients. The CNN delineated the vast majority of lymphoma manifestations while avoiding physiological uptake with only minimal further adjustments necessary

**Fig. 6:**
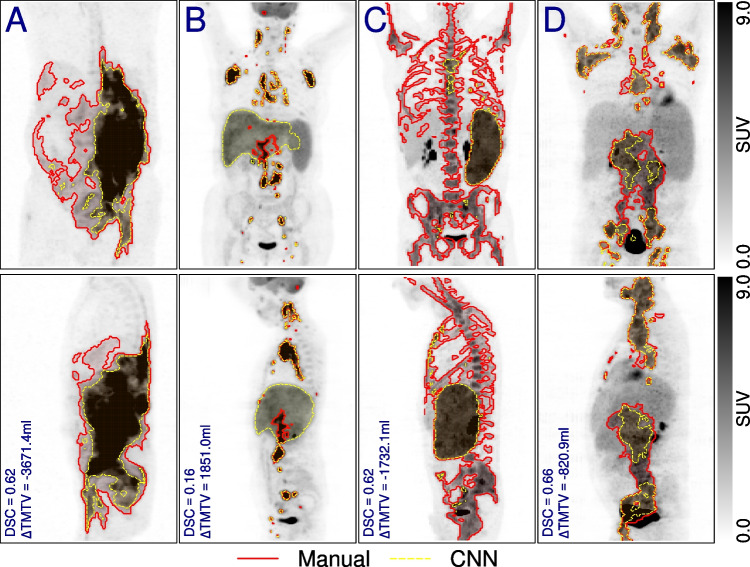
Examples of the outliers in Fig. 2 from the cross-validation experiment. These studies represent the most frequent reasons for large deviations in TMTV between automated and manual delineations. Patient **A**: diffuse intestinal and mesenterial uptake; patient **B**: false inclusion of liver with elevated [^18^F]FDG uptake; patient **C**: false negative bone uptake; patient **D**: partial omission of diffuse uptake in confluent retroperitoneal lymphoma. Coronal (top) and sagittal (bottom) maximum intensity projections of PET images are shown

Examples of successful and unsatisfactory (top 1% outliers according to |ΔTMTV| values) delineations from the cross-validation data are shown in Figs. 5 and 6, respectively. The examples in Fig. 5 illustrate that the CNN is able to handle bulky disease and to correctly identify almost all lesions, even in complex delineation tasks, while also excluding physiological tracer uptake. On the other hand, the outliers shown in Fig. 6 indicate that some delineation tasks still pose a challenge to the present CNN, even though the number of severe outliers was rather small (see Fig. 2). Typical challenging cases include diffuse intestinal and mesenterial uptake, potential liver or spleen involvement, bone marrow infiltration, and diffuse retroperitoneal uptake (see patients A-D in Fig. 6, respectively).

**Fig. 7:**
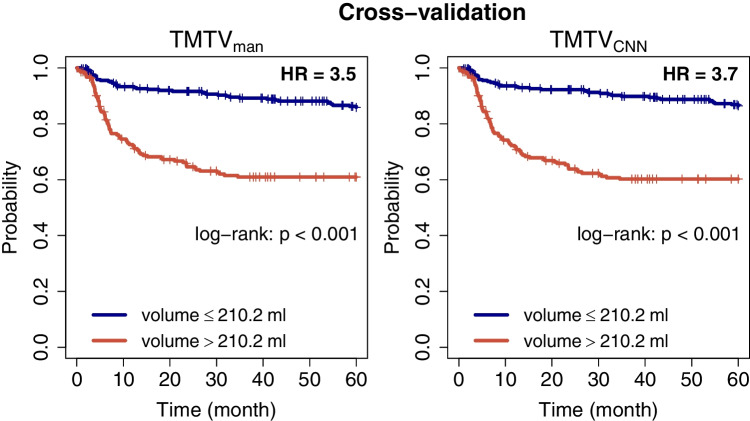
Kaplan-Meier curves with respect to PFS in cross-validation data (*N* = 534). The threshold for group separation was optimized for manual delineation and applied globally

### Survival analysis

Univariate Cox regression analysis in the cross-validation data revealed TMTV_man_ as well as TMTV_cnn_ as highly prognostic factors for PFS with very similar hazard ratios (HR = 3.5; *p* < 0.001 vs. HR = 3.7; *p* < 0.001, respectively). Binarization resulted in a different classification in only 3.6% of the patients, where classification was incorrect in 2.1% of the cases for TMTV_man_ and in 1.5% of the cases for TMTV_cnn_. Corresponding Kaplan-Meier curves are shown in Fig. 7.

## Discussion

In this investigation, we trained a neural network to delineate all lymphonodal and extralymphonodal manifestations in patients with Non-Hodgkin lymphoma using combined [^18^F]FDG-PET/CT data. The training data were acquired within the multicenter PETAL trial covering a large variety of disease subtypes and imaging conditions encountered in clinical routine. The employed ground truth generation procedure did not rely on the fixed SUV thresholds. The ground truth delineations included very small and low uptake lesions, and were generated iteratively with assistance of the intermediate CNNs, which summarizes the main innovations of this work. Comparison between manual and CNN-based delineations revealed overall good spatial concordance between them. We achieved an aggregated DSC = 0.895, median DSC = 0.919, and the average DSC = 0.802 ± 0.269 in the main dataset, which is comparable to aggregated DSC = 0.886 in [25], DSC = 0.861 in [29], and DSC = 0.846 ± 0.002 in [30], median DSC = 0.71 to 0.90 (depending on the post-processing and the reference delineation) in [27] and considerably better than the mean DSC of 0.68 ± 0.12 in internal testing in [24]. In terms of volumetric concordance, our automatically extracted TMTVs were highly correlated to the manually derived ones with *R*^2 ^= 0.974, which is higher than *R*^2^ = 0.88 in cross-validation in [26] and *R*^2^ = 0.89 in internal testing in [24]. The achieved Spearman correlation *ρ *= 0.965 is in line with *ρ* = 0.917 to 0.982 in [27] and *ρ *= 0.97 in [25], and higher than *ρ* = 0.878 in [28] and *ρ* = 0.76 in [21]. One should, however, exercise caution when comparing results across different publications and keep in mind the heterogeneity in datasets, delineation approaches, and evaluation methodologies. For example, the outlier exclusion had a dramatic effect on the $R^2$ metric in our work (*R*^2^ = 0.829 before the exclusion, rapidly growing up with every excluded case, see supplementary Fig. S3) due to the presence of extreme outliers, as can be seen in Fig. 2. Such outliers have disproportionally high effect on the results of correlation analysis [42] making them not representative (or even misleading) of the properties of the vast majority of the data. Other publications do not employ the same strategy for the *R*^2^ calculation which might partially explain the observed gap in reported performance. These studies, however, do not contain such extreme isolated outliers, and therefore we expect the effect of exclusion of a small number of the most deviant data points to be limited there.

In external validation, the DSC was notably lower (aggregated DSC = 0.715), which can be partly explained by the different delineation strategies. In [18], lesion delineation was essentially performed by application of an SUV = 4 threshold, followed by exclusion of small lesions (with volume < 3 ml) and manual interactions. For the ground truth generation in the current study, a lesion-wise or even voxel-wise threshold was applied to account for partial volume effects and lesion heterogeneity. Consequently, the trained network performs the delineation in a similar manner. This can have a strong effect on the DSC and on the TMTV, especially in patients with low total lesion burden. Furthermore, the SUV ≥ 4 and the lesion volume ≥ 3 ml constraints used for the ground truth definition in the external dataset (some small lesions were added manually, but only when deemed necessary and practical) lead to the omission of lesions which were included with our method, as can clearly be seen in Table 3. Finally, the external dataset included Hodgkin lymphoma patients who were not part of our training dataset and also had studies with challenging delineations intentionally overrepresented.

To at least subjectively assess the delineation accuracy in the external dataset, an independent observer (IA) with over 20 years of experience, not involved in ground truth generation, reviewed manual and automated delineations for all 60 patients. The observer was blinded to the method used for producing the delineations, and their presentation order was randomized. The goal was to rate both delineations — on a scale from 1 to 5 (5 is the best) — if they were correct within our delineation strategy (i.e. inclusion of all pathological uptake). Remarkably, both delineations received an identical average score of 3.68. In 20 patients, the CNN produced a better delineation, in 20 patients, the manual delineation was considered better, and in 20 patients, the scores were equal. Examples of discordant manual and automated delineations in the external dataset are shown in the supplementary Fig. S4. Although this analysis cannot be considered comprehensive, as the important clinical information about the patients was not available to the independent observer, it nevertheless suggests that, at least qualitatively, the presented delineations were on par.

Overall, our results underline an inherent general problem of comparing delineations performed using different strategies in the absence of a definitive gold standard. In the current study, the delineation was focused on providing the best possible ground truth for the CNN training (at the expense of being very time-consuming), while the focus in the benchmark dataset was on using the most reproducible semi-automatic procedure which is fast enough to be used in a clinical context. The typically employed numerical agreement metrics are not representative of the delineation quality in such cases, necessitating the use of subjective scores.

The largest deviations of CNN-based delineation from the ground truth were found for diffuse manifestations in spleen, liver, bone marrow, and gastrointestinal tract. This was also observed in the main dataset, but since data of these types are overrepresented in the benchmark dataset, such discrepancies have a stronger effect on evaluation metrics there. For liver and bone marrow infiltration, this indicates a rather poor performance of the network, which can be at least partly explained by the low number of corresponding PET-positive cases in the training data (*N* = 5 and *N* = 7, respectively). It can be expected that when including more data of this type, the network performance will improve. Gastrointestinal manifestations were much more frequent (*N* > 50), and in general, the network performed very well for such data, with few exceptions only. Similarly good results were achieved for the spleen. Overall, 29 infiltrations of the spleen were present in the main data, from which 27 were correctly identified by the CNN and two were missed. Only in three cases, the CNN produced false-positive spleen delineations. It should be noted that even though [^18^F]FDG-PET is very capable in detecting bone marrow [43] or splenic involvement of aggressive lymphoma, there is a well-known diagnostic gap when it comes to diffuse elevated uptake, which is generally considered negative, regardless of its intensity [44]. The possible masquerade of manifestations in this context remains a clinical dilemma not only in the bone marrow but also in the spleen, which leads to a large inter-observer variability [18]. Nevertheless, the presence of strong outliers — however rare they are — emphasizes the need for visual verification of the produced delineations and reaffirms the role of the CNN as a delineation assistance tool rather than a fully automatic replacement for human observers.

The overall good performance of the network consequently translates into a good concordance in risk stratification based on TMTV_man_ and TMTV_cnn_, respectively, as Fig. 7 shows. The risk assessment performed with the two methods differed only in 3.6% of the patients. However, it is worth mentioning that this is also a consequence of the here-applied two-risk model with a single cutoff. Only when TMTV_man_ is below the cutoff and TMTV_cnn_ is above the cutoff (or vice versa), risk stratification changes. Even for very large ΔTMTV, stratification might be unaltered in cases where both volumes are above the cutoff, as was the case in, e.g., Fig. 6A. This might lead to the impression that accurate delineation is not very important besides some exceptions. However, the required accuracy obviously increases when a multi-risk model with several cutoffs is applied. To ensure consistency of such models across different centers and scanner hardware, a delineation method capable of accounting for varying image resolution and involving small lesions is needed. These requirements conditioned our approach to the ground truth generation.

A preliminary assessment of the impact of small lesions showed that they can notably change TMTV. As can be seen in Fig. 3, already for a minimum lesion volume of 1.5 ml, the difference between TMTV with and without such lesions can exceed 50 ml. On the other hand, if this difference results in a significant effect on therapy outcome prediction or therapy response assessment with a single- or a multi-cutoff model is an open question. This has to be addressed in a detailed survival analysis, which is beyond the scope of the current investigation. However, until this question is finally answered, it is recommended to also include small lesions in the determination of TMTV, especially for patients with low to moderate total tumor burden. The presented CNN can help to reduce the time needed to perform this task, but it does not fully solve this problem. In the detection of lesions with a volume below 1 ml, we achieved only a TPR of 56.0% in the main dataset and 63.9% in the validation dataset. Nevertheless, even with the current performance level, the CNN assistance proved to be indispensable in generating the ground truth delineations in the present study. The endeavor of accurate contouring of almost 9000 lesions would be otherwise infeasible with semi-automatic methods alone. With our hardware, the CNN execution takes 1.5–2 minutes per scan for the majority of the patients in the external dataset with the maximum of 2.5 minutes. Batch processing of all 60 patients in the external dataset took only 45 minutes due to parallelization of operations as well as avoiding the need to repeatedly load and initialize the CNN for each individual patient. This time, however, does not need to be actively spent by the specialist. In principle, the TMTV generation can be performed prior to the visual inspection of the patient or even as a part of automated workflow right after the image reconstruction.

The present study has several limitations. First of all, the images in the main dataset were acquired before 2014, i.e. no data from state-of-the-art digital PET scanners were included in the training. Images from newer systems, especially long axial field-of-view scanners, feature overall higher resolution and lower noise levels than those in the included data. This shift in image characteristics might potentially hamper the performance of the CNN when applied to newer data. Another limitation is the above-mentioned relatively low number of patients with infestation of liver and bone marrow. We are currently collecting more data of both types, i.e. data from state-of-the-art machines as well as data with liver and bone involvement, with the goal to enhance the training dataset and update the network model once a sufficient number of additional datasets is collected. An alternative method to achieve generalization to the future generation of scanners — and allow for easier generation of the ground truth delineations — is harmonizing of the input image properties (see, e.g., EARL accreditation and harmonization initiatives [45]). The evidence on the effectiveness of this strategy for DL-based lesion delineation in PET is, however, limited so far. Additional limitation concerns the disease subtype composition in the training dataset: only non-Hodgkin lymphoma was included with the majority of the data being DLBCL. Therefore, caution needs to be exercised when applying the CNN to more rare subtypes, such as follicular lymphoma, or to Hodgkin lymphoma. Further limitations are related to the labeling process. Only one physician was involved in the generation of the manual delineations. Due to the lack of objective ground truth and the challenging nature of the task, delineation should ideally be performed by a collective of physicians. However, it was not feasible due to the sheer amount of included data and the associated high time requirements for ground truth generation. Finally, the ground truth delineations were produced with the assistance of intermediate CNNs, which could make them more similar to the final network outputs compared to the purely manual or semi-automatic delineations and, therefore, positively bias the evaluation metrics. We do not expect this effect to be substantial since the manual corrections were applied vigilantly whenever deemed necessary, but it cannot be excluded completely. This incentive is indirectly reinforced by independent subjective evaluation of the produced delineations in the external data, showing comparable quality of automated and manual delineations within our delineation strategy.

## Conclusion

In this study we presented a CNN model capable of accurate TMTV delineation in [^18^F]FDG-PET/CT images of lymphoma patients. Unlike other existing deep-learning-based solutions, our network is trained to delineate all tumor lesions, regardless of their size or uptake characteristics, while accounting for effects of limited image resolution. Although further improvements are possible, already now, the developed neural network allows for substantial acceleration of quantitative analysis of lymphoma imaging data and has the potential for supervised clinical use. Inclusion of small lesions will facilitate investigation of their impact on therapy outcome prediction and response assessment in further studies.

## Supplementary Information

Below is the link to the electronic supplementary material.

Supplementary file 1 (pdf 915 KB)

## Data Availability

The network models developed in the present study, along with detailed usage instructions, are publicly available at https://github.com/hzdr-MedImaging/LyROI.
