# Supplementary material for: A convolutional neural network for fully automated total metabolic tumor volume delineation in patients with aggressive Non-Hodgkin lymphoma
Source: Eur J Nucl Med Mol Imaging. 2026 Mar 24;53(8):5074–87. doi: 10.1007/s00259-026-07810-9 (PMC13249771; doi:10.1007/s00259-026-07810-9)
Supplement: Supplementary file 1 — (pdf 915 KB) [file 259_2026_7810_MOESM1_ESM.pdf]

European Journal of Nuclear Medicine and Molecular Imaging

Supplementary Material:

A convolutional neural network for fully automated total metabolic tumor volume delineation in patients with aggressive Non-Hodgkin lymphoma.

Pavel Nikulin [1,*,†], Sebastian Hoberück [2,*], Ivayla Apostolova [3], Jens Maus [1], Andreas Hüttmann [4], Ulrich Dührsen [4], Frank Kroschinsky [5], Jörg Kotzerke [2], Malte von Bonin [5], Ralph A. Bundschuh [2,6,7,8], Anja Braune [1,2,9], Frank Hofheinz [1]

[1] *Helmholtz-Zentrum Dresden-Rossendorf, Department of Positron Emission Tomography, Institute of Radiopharmaceutical Cancer Research, Dresden, Germany*

[2] *Department of Nuclear Medicine, University Hospital Carl Gustav Carus, Technische Universität Dresden, Dresden, Germany*

[3] *Department for Diagnostic and Interventional Radiology and Nuclear Medicine, University Hospital Hamburg-Eppendorf, Hamburg, Germany*

[4] *Department of Hematology, West German Cancer Center, University Hospital Essen, University of Duisburg-Essen, Essen, Germany*

[5] *Dresden University Hospital, Medical Department I, Dresden, Germany*

[6] *Helmholtz-Zentrum Dresden-Rossendorf, Institute of Radiopharmaceutical Cancer Research, Dresden, Germany*

[7] *German Cancer Consortium (DKTK), Partner Site Dresden, Dresden, Germany*

[8] *National Center for Tumor Diseases (NCT), NCT/UCC Dresden, a partnership between DKFZ, Faculty of Medicine and University Hospital Carl Gustav Carus, TUD Dresden University of Technology, and Helmholtz-Zentrum Dresden-Rossendorf, Germany*

[9] *Carl Gustav Carus Faculty of Medicine, Technische Universität Dresden, Dresden, Germany*

Iterative ground truth generation

In the iterative network training approach, an initial training was done with only a small subset of the available data, and the subsequently developed models assisted in the labeling of further training data. The ground truth for the initial network training was generated by delineating all PET-positive lesions in $N = 26$ PET/CT images with one of the following methods depending on the tracer uptake of the lesion:

[*] P. Nikulin and S. Hoberück contributed equally to this article.

[†] Bautzner Landstrasse 400, 01328 Dresden, Germany. E-mail: p.nikulin@hzdr.de

- High and homogeneous uptake: global adaptive threshold [*1*]
- High and heterogeneous uptake: local adaptive threshold [*2*]
- Low or diffuse tracer accumulation: manually adjusted global fixed threshold,

where *global threshold* refers to a single threshold for each region of interest (ROI) and *local threshold* refers to an individual threshold for each voxel in each ROI. All threshold-based delineations were visually inspected and corrected by manually adding or removing voxels as deemed necessary. In the following, we refer to this type of lesion delineation as *manual delineation*. The initial images were selected according to the following criteria: not more than 10 lesions per patient and the presence of notable non-pathological tracer uptake (heart, kidneys, bladder, etc.). After initial training with these data, the iteration was performed as follows:

1. Inference of the current network model on a subset of the data which was not included in previous network trainings so far.
2. Correction of the delineations with the above-described methods in data in which the estimated time for correction was below 5 minutes.
3. Inclusion of the corrected data in the training dataset and complete re-training of the network.

Steps 1 to 3 were repeated until all available data were included in the training. The complete sequence of the training set sizes in each iteration was as follows: $N = 26$, 79, 183, 272, 383, 506, 641, 690, 775, 859, 978, 1133, 1192. Manual delineations were performed using the software `ROVER` (version 3.0.78, ABX GmbH, Radeberg, Germany) by a tandem of a nuclear medicine physician (SH) with over 10 years of experience in oncological PET and a physicist (FH) with over 20 years of experience in development of delineation algorithms in PET.

The progress of iterative network training is illustrated in fig. S1. Shown are the results of a CNN-based delineation corresponding to four different training set sizes ($N = 26$, 183, 383, and 775). Note, that the patient data displayed in the figure was not included in any of these training sets. On the left, the result after application of the initial network is shown. Most of the lesions were missed by this early CNN. The results became clearly better with the progression of the iterations. After application of the CNN trained with $N = 775$ datasets, the produced delineation was good enough for it to be manually corrected within a 5-minute time frame, and thus was subsequently included in the training dataset.

Network training

The network design and training were performed with `nnU-Net` (version 2.5) [*3*] software framework for `Python` (version 3.10) based on `PyTorch` (version 2.3.1) deep learning library. The framework has a CNN with a U-Net-like architecture at its core. `nnU-Net` features strong self-configuration capabilities, allowing both the network architecture and the data preprocessing steps to automatically adapt to the training data. The resulting configuration can be, however, influenced by choosing one of the configuration presets (affects the variant of U-Net architecture and the target GPU memory) or by manually

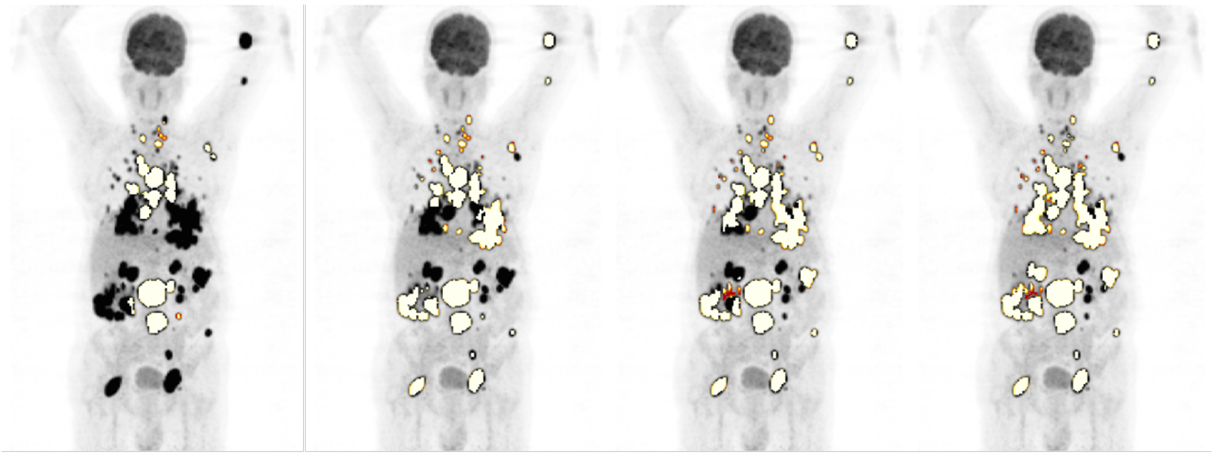

FIGURE S1: Illustration of the iterative network training for a patient outside of the training dataset. Shown are coronal from anterior maximum intensity projections of PET images. Bright yellow color indicates automatically delineated ROIs and the value N below each column shows the number of training samples used to develop the corresponding CNN model.

editing the configuration files or source code. Preprocessing and training procedures can be influenced in a similar way.

Three configurations of the `nnU-Net` were used for developing models in each iteration: regular U-Net, medium residual encoder U-Net (8 GB GPU memory target), and large residual encoder U-Net (24 GB GPU memory target). The last configuration was skipped in the initial iteration due to the limited amount of training data. The training was done using Cross-Entropy + Dice loss function with the smoothing parameter in the Dice term set to 0. The smoothing term is intended to provide numerical stability for the Dice calculations with small structures. However, we observed the opposite effect, at least in application to cancer lesion delineation in PET. In our preliminary experiments, the default loss function was causing frequent failures in the training procedure, necessitating the aforementioned change. Additionally, the `batch size` was set to 3 instead of the self-configured value of 2 during the iterative training. This setting was giving slightly better results during the visual examination of the results in the early iterations and was kept the same for the whole procedure for consistency. The choice of this parameter was reassessed with the final dataset and it was reverted to `batch size` = 2 for the final evaluation. Among other things, the `batch size` affects foreground oversampling rate at low batch sizes, which, in turn, translates into a different balance between sensitivity and positive predictive value for detecting tumor voxels. `Batch size` = 2 leads to a higher oversampling rate than `batch size` = 3 (50% vs. 33%, respectively), leading to higher sensitivity values, which is desirable in the present application. During the iterative training procedure, all processed data were used for training in each step. In the final iteration, the data were split into training and testing subsets in a proportion of 80/20% for the 5-fold cross-validation procedure (more details in the manuscript). Other `nnU-Net` settings were left at default values.

The network architectures corresponding to all three `nnU-Net` configurations in the final itera-

tion are depicted in fig. S2. The main building element of the regular U-Net is the convolution block consisting of $3 \times 3 \times 3$ convolution, instance normalization, and leaky ReLU activation. The downsampling is performed via a convolution block with a strided convolution (`stride` $= 2 \times 2 \times 2$ or $1 \times 1 \times 2$), and the upsampling is done via $3 \times 3 \times 3$ transposed convolutions. The number of convolution blocks in the encoder and the decoder of the U-Net are approximately the same. In contrast, the residual encoder U-Net shifts the main computational load towards the (residual) encoder. The main building element of the residual encoder is the residual convolution block, comprising two consecutive convolution blocks and the residual connection linking the input of the whole block with the last leaky ReLU for improved gradient flow. The number of consecutive residual block applications grows after each downsampling operation (capped at 5): 1, 2, 3, 5, 5, 5 repetitions at the first to the sixth resolution stages, respectively. Downsampling is performed via the residual blocks with the strided first convolution (`stride` $= 2 \times 2 \times 2$ or $1 \times 1 \times 2$) and the average pooling for downsampling in the residual pathway. For more details on these architectures, see [*4*].

Before applying the CNN, all images were resampled to $4.06 \times 4.06 \times 3.00$ mm^3 voxel size and normalized using the default `nnU-Net` normalization schemes for each modality. The matrix size for the input image patches was set to $112 \times 112 \times 192$ (corresponding to $45.5 \times 45.5 \times 57.6$ cm^3 coverage) for the regular U-Net and medium residual encoder U-Net, and to $160 \times 160 \times 288$ (corresponding to $65.0 \times 65.0 \times 86.4$ cm^3 coverage) for the large residual encoder U-Net. In all configurations, the number of feature channels started at 32 and increased by a factor of 2 after each downsampling operation (capped at 320).

The neural network training was conducted on a system equipped with four NVIDIA A100/SXM GPUs, each with 80 GiB of graphics memory. The system was running Ubuntu Linux 24.04.3 LTS and CUDA 12.4 with NVIDIA driver version 550.163.01 in place.

Outlier handling

The sensitivity of the correlation analysis to the outliers is a well known issue. Data points with high leverage disproportionally affect the value of R^2 metric and the results of linear regression [*5*]. To provide more fair estimate of the R^2 for the vast majority of the patients, we exclude top 1 percentile of data exhibiting the largest deviations in terms of TMTV.

In order to show systematically that the excluded points are indeed the outliers with disproportionate influence on the correlation and regression analysis, we analyzed the changes in R^2 metric and in the linear regression slope with exclusion of different percentiles of the most deviant data points. The results of this analysis are presented in fig. S3. As can be seen, the R^2 changes rapidly in the $[0,1]\%$ interval, and it continues to change noticeably in the $[1,2]\%$ interval. R^2 stabilizes afterwards and increases gradually with further exclusion of the data as expected. Similar behavior can be observed for the linear regression slope parameter. This indicates that around 1% to 2% of the data points disproportionately affect the analysis results which can be attributed to them being outliers, as explained above. Here, we opted for more conservative value of 1% for a cutoff to avoid overly optimistic reporting.

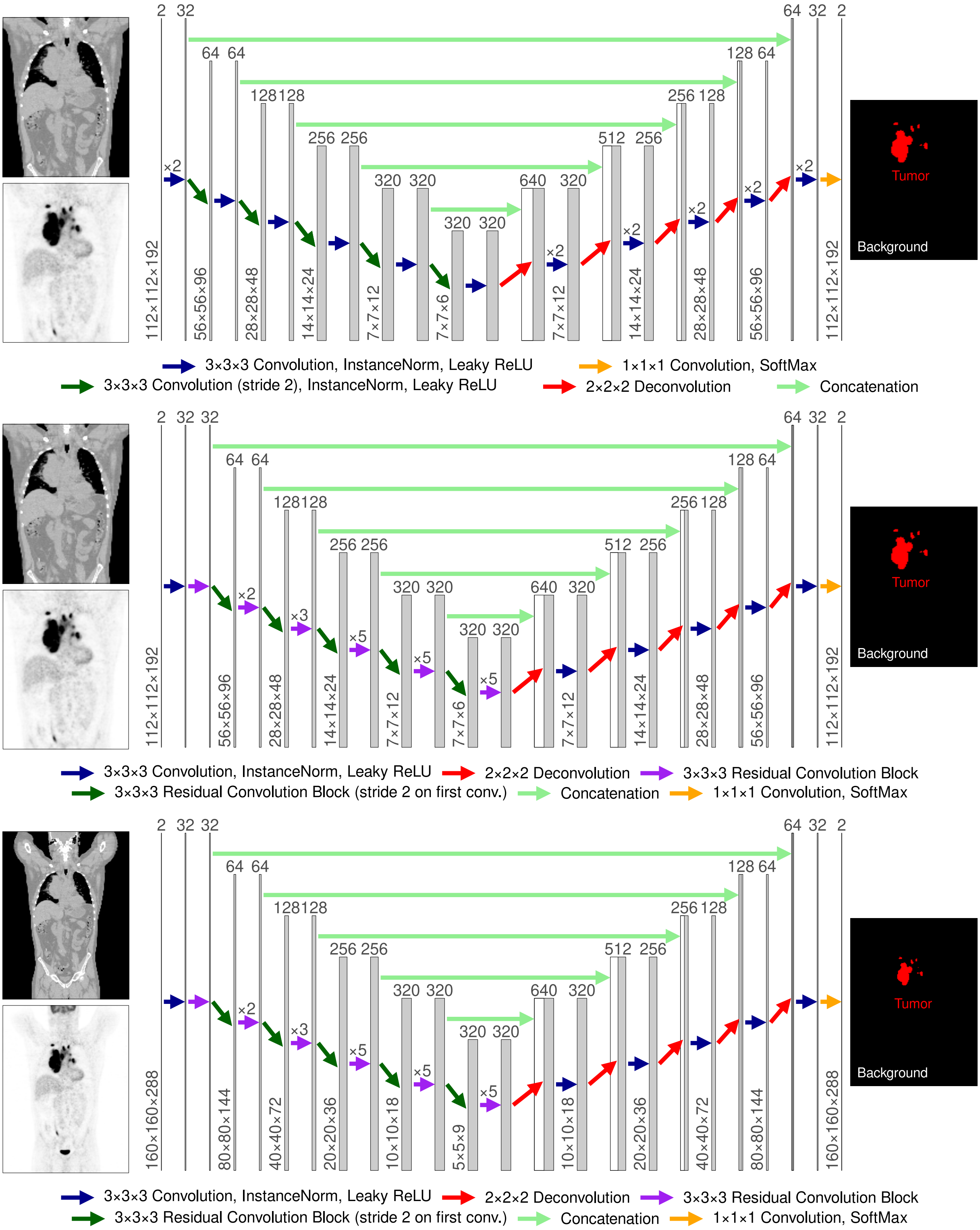

FIGURE S2: Final architectures for three `nnU-Net` configurations: regular U-Net (top), medium residual encoder U-Net (middle), and large residual encoder U-Net (bottom). Boxes depict tensors and arrows depict operations. Numbers above tensors show the number of feature maps and numbers on their side show spatial dimensions. Numbers above some operations indicate that that operation is repeated multiple times in a row. Panels on the left and on the right depict example inputs (CT and PET) and the outputs of the networks, respectively.

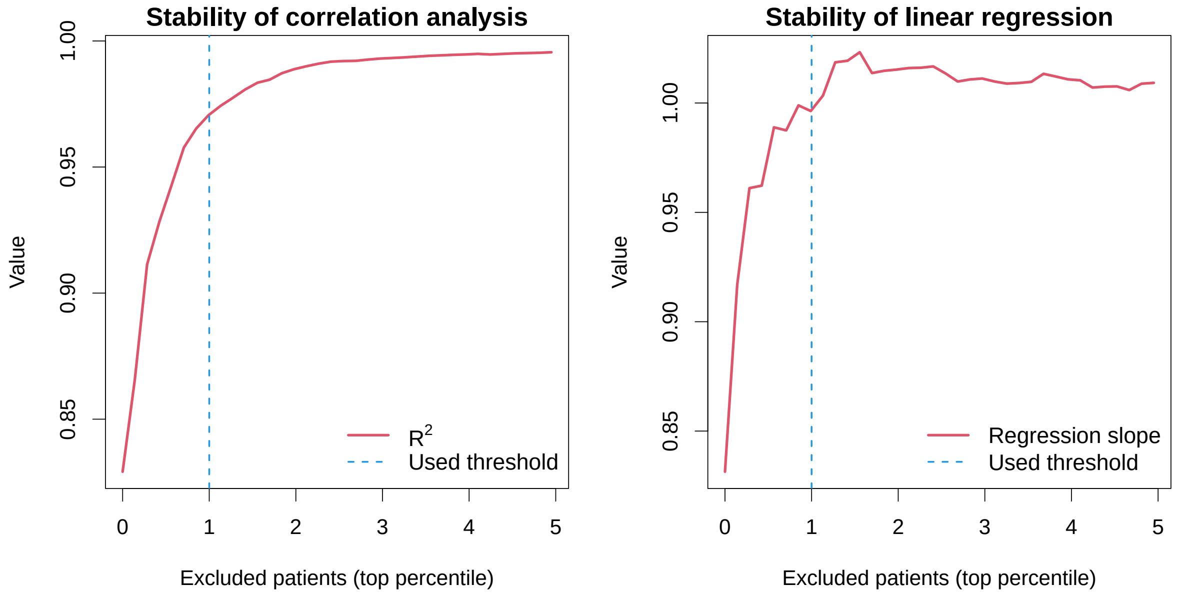

FIGURE S3: The change in the R^2 metric and in the linear regression slope with exclusion of different percentiles of the most deviant (with respect to the TMTV) data points.

Examples (external data)

Fig. S4 shows examples of discordant manual and automated delineations in the external dataset. These delineations were rated by an external observer (IA) as follows (manual/automated): A: 3/5; B: 2/4; C: 4/3; D: 5/3, where 5 is the highest and 1 is the lowest score. I.e., for studies A and B, the automated delineation was rated higher, and for studies C and D, the manual delineation was rated higher. In A, B, and C, the CNN has identified more tumor lesions, most of which had diffuse uptake below or slightly above the SUV = 4 threshold. In C, however, the diffuse splenic uptake was not included in the automated delineation, leading to a big difference in TMTV values between the two delineations and the corresponding low score. Finally, in D, the CNN has missed several obvious lesions included in the manual delineation.

Interestingly, in study B, despite the fact that automated delineation included more lesions than the manual one, the $TMTV_{cnn}$ was actually lower than $TMTV_{man}$ by more than 400 ml. This can be explained by the more accurate contouring of the bulky lesion by the CNN with exclusion of the background areas affected by the spill-over from the high-accumulating tumor due to the partial volume effect. This example demonstrates that the effect on TMTV calculation from the contouring approach alone can be substantial, especially in bulky, heterogeneous, and highly accumulating lesions, emphasizing the flaws of fixed-threshold based delineation methods.

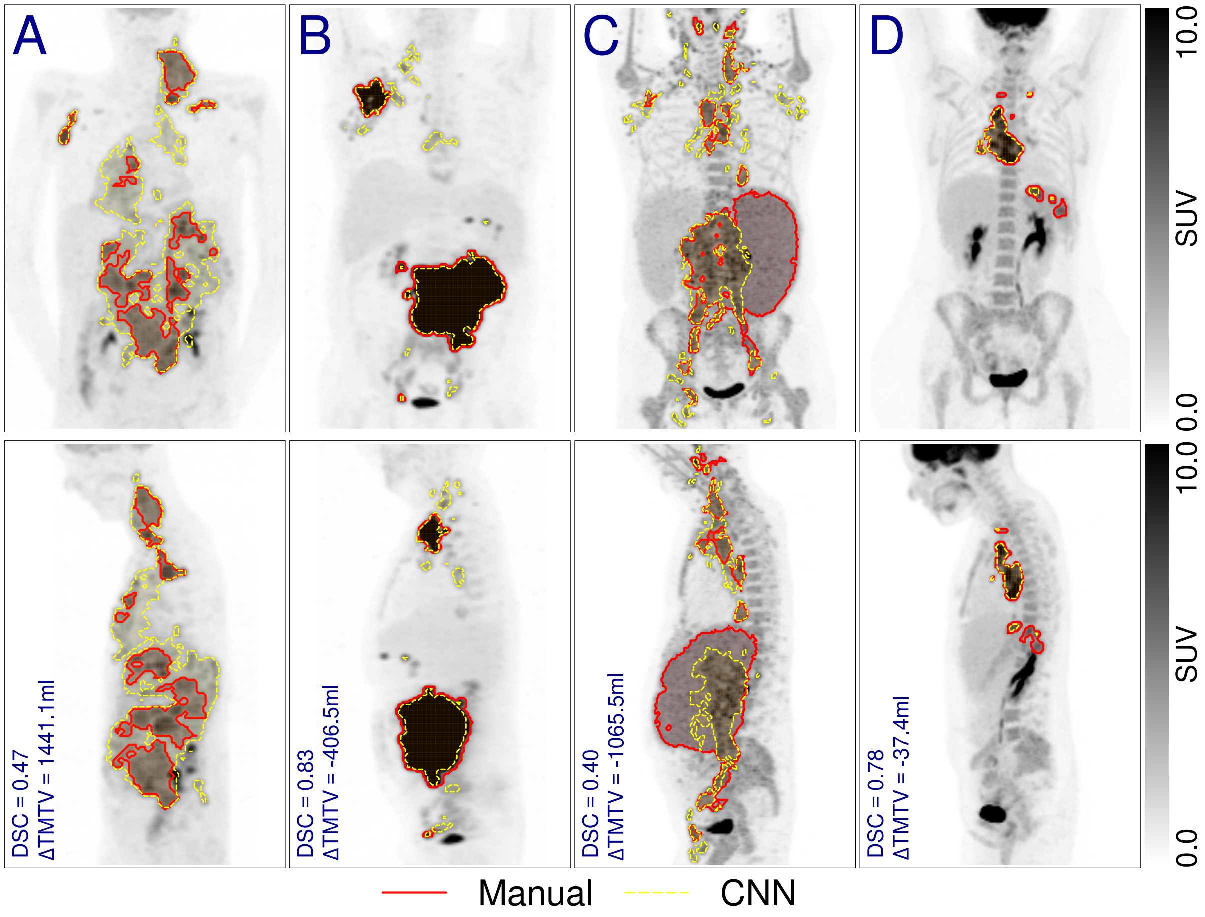

FIGURE S4: Visualization of discordant manual and automated delineations in four exemplary studies (A–D) from the external benchmark dataset. Shown are coronal from anterior (top) and sagittal (bottom) maximum intensity projections of PET images of Hodgkin (A & D), diffuse large B-cell (B), and follicular (C) lymphoma patients. For studies A and B, the automated delineation was rated higher, and for studies C and D, the manual delineation was rated higher by the independent observer.

TABLE S1: Histology, IPI staging, and Therapy. Note that this information was only available for patients which were actually included in the PETAL trial.

Characteristics	Value (%)
**Histology**	
DLBCL	371 (72)
B-cell	50 (9.7)
T-cell	38 (7.4)
FL	25 (4.9)
Violation I/E	31 (6)
**IPI staging**	
n.a.	3 (0.6)
0	65 (12.6)
1	122 (23.7)
2	138 (26.8)
3	118 (22.9)
4	53 (10.3)
5	16 (3.1)
**Therapy after interim PET**	
4xR-CHOP	238 (46.2)
4xR-CHOP +2xR	218 (42.3)
6xR-CHOP	30 (5.8)
6xB-ALL	29 (5.6)

Histology DLBCL: diffuse large B-cell lymphoma; B-cell: other large and borderline B-cell lymphoma; T-cell: T-cell lymphoma; FL: follicular lymphoma grade 3; Violation I/E: violation of inclusion or exclusion criteria. **Therapy** All patients received 2 cycles of the CHOP regimen (plus rituximab for CD20-positive lymphomas) followed by interim PET scanning. Depending on the result of the interim scan, the patients were then allocated to one of the treatments indicated. CHOP regimen: cyclophosphamide, doxorubicin, vincristine, and prednisone; R: rituximab; B-ALL: Burkitt protocol.

TABLE S2: Delineation performance in cross-validation data without exclusion of the negative scans as measured by different metrics. N indicates the number of images included in the analysis.

Metric	Aggregated	Mean ± SD	Median	IQR
Cross-validation w/o excl. ($N = 1192$)				
DSC	0.889	0.733 ± 0.380	0.940	[0.658, 1.000]
TPR	0.896	0.487 ± 0.453	0.628	[0.000, 0.959]
PPV	0.882	0.497 ± 0.461	0.719	[0.000, 0.959]

TABLE S3: TMTV determination performance in cross-validation data without exclusion of the negative scans as measured by different metrics. N indicates the number of images included in the analysis.

Metric	Cross-validation w/o excl. ($N = 1192$)		
	Mean ± SD	Median	IQR
$TMTV_{man}$ (ml)	179.5 ± 467.6	3.0	[0.0, 139.9]
$TMTV_{cnn}$ (ml)	179.8 ± 430.3	7.5	[0.0, 146.2]
ΔTMTV (ml)	0.4 ± 185.5	0.0	[-0.8, 1.9]
$\lvert\Delta TMTV\rvert$ (ml)	31.3 ± 182.9	1.4	[0.0, 9.9]

REFERENCES

[1] Hofheinz F, Pötzsch C, Oehme L, Beuthien-Baumann B, Steinbach J, Kotzerke J, et al. Automatic volume delineation in oncological PET. Evaluation of a dedicated software tool and comparison with manual delineation in clinical data sets. Nuklearmedizin. 2012;51:9–16.

[2] Hofheinz F, Langner J, Petr J, Beuthien-Baumann B, Steinbach J, Kotzerke J, et al. An automatic method for accurate volume delineation of heterogeneous tumors in PET. Med phys. 2013; 40(8):082503.

[3] Isensee F, Jaeger PF, Kohl SAA, Petersen J, Maier-Hein KH. nnU-Net: a self-configuring method for deep learning-based biomedical image segmentation. Nature Methods. 2020;18(2):203–211. doi: 10.1038/s41592-020-01008-z.

[4] Isensee F, Maier-Hein KH. An attempt at beating the 3D U-Net. 2019. doi:10.48550/ARXIV.1908.02182.

[5] Devlin SJ, Gnanadesikan R, Kettenring JR. Robust estimation and outlier detection with correlation coefficients. Biometrika. 1975;62(3):531–545. doi:10.1093/biomet/62.3.531.

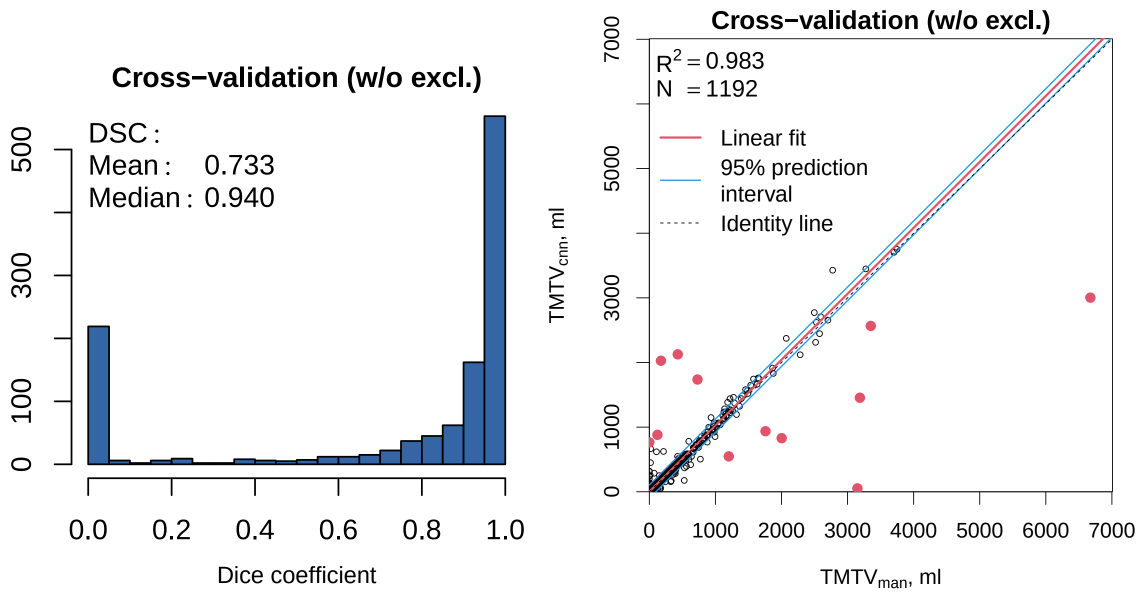

FIGURE S5: Frequency distribution of the observed Dice coefficients (left) and a correlation between manually and automatically derived TMTV (right) in the cross-validation without exclusion of the negative scans ($N = 1192$ images). Solid red points indicate outliers, defined as data points where the deviation of CNN from manual delineation exceeds the 99% percentile (i.e. the top 1%). These outliers were excluded from the regression analysis. The red line represents the least squares fit of a straight line to the remaining data. The blue lines delineate the corresponding 95% prediction (tolerance) interval of the expected scatter of individual data points around the regression line.
